# A Quantitative Evaluation of Drive Pattern Selection for Optimizing EIT-Based Stretchable Sensors

**DOI:** 10.3390/s17091999

**Published:** 2017-08-31

**Authors:** Stefania Russo, Samia Nefti-Meziani, Nicola Carbonaro, Alessandro Tognetti

**Affiliations:** 1 Autonomous System and Robotics Research Centre, University of Salford, Manchester M5 4WT, UK; s.nefti-meziani@salford.ac.uk; 2 Research Centre E. Piaggio, University of Pisa, 56122 Pisa, Italy; nicola.carbonaro@centropiaggio.unipi.it (N.C.); a.tognetti@centropiaggio.unipi.it (A.T.); 3 Department of Information Engineering, University of Pisa, 56122 Pisa, Italy

**Keywords:** stretchable sensor, conductive fabric, inverse problem, performance parameters

## Abstract

Electrical Impedance Tomography (EIT) is a medical imaging technique that has been recently used to realize stretchable pressure sensors. In this method, voltage measurements are taken at electrodes placed at the boundary of the sensor and are used to reconstruct an image of the applied touch pressure points. The drawback with EIT-based sensors, however, is their low spatial resolution due to the ill-posed nature of the EIT reconstruction. In this paper, we show our performance evaluation of different EIT drive patterns, specifically strategies for electrode selection when performing current injection and voltage measurements. We compare voltage data with Signal-to-Noise Ratio (SNR) and Boundary Voltage Changes (BVC), and study image quality with Size Error (SE), Position Error (PE) and Ringing (RNG) parameters, in the case of one-point and two-point simultaneous contact locations. The study shows that, in order to improve the performance of EIT based sensors, the electrode selection strategies should dynamically change correspondingly to the location of the input stimuli. In fact, the selection of one drive pattern over another can improve the target size detection and position accuracy up to 4.7% and 18%, respectively.

## 1. Introduction

Electrical Impedance Tomography (EIT) is an imaging method in which electrodes placed at the periphery of a conductive body are used to both perform a small current injection and measure the resulting induced voltages; then, an inverse reconstruction algorithm is applied and an image of the internal conductivity distribution of the body is shown.

EIT is mainly applied in clinical applications for patient monitoring [1] and pulmonary and cardiac functionality [2]. This is because EIT is a non invasive and non-ionizing method, different from other medical imaging techniques. Other applications include damage detection [3] and pressure sore prevention [4].

Recent studies [5] have focused on the application of EIT techniques on thin, stretchable and piezoresistive materials in order to create pressure sensitive sensors.The materials used for EIT-based sensors exhibit a change in their internal resistance when a mechanical solicitation is applied. Therefore, an image of the pressure input can be reconstructed from the boundary voltages.

In contrast to EIT pressure sensitive sensors, most available flexible sensors have rigid sensing materials that are enclosed in soft substrates [6,7,8], and despite being flexible, they are still prone to failure when mounted over curved surfaces due to the presence of different internal structures that create fragility. Thus, EIT-based sensors are advantageous since they do not have that rigid network within the active sensing area. This makes them inherently stretchable; therefore, they can be placed over surfaces with different topologies without losing their functionality. For example, EIT-based sensors can be used for developing wearable devices for human users as seen in [9,10]. In addition, a study on tomographic methods for distributed strain sensing in PU-based nanocomposites can be found in [11]. However, the drawback to these sensors is their low spatial resolution. This is because they are considered as inverse problems as described in [12]. Thus, EIT systems are mathematically severely ill-posed, nonlinear and are very sensitive to small changes in potential at the boundary measurements. This results in low spatial resolution that jeopardizes their potential application as sensors.

With the aim of compensating for drawbacks that are present in EIT systems, previous works [13,14,15] have demonstrated that the spatial resolution of an EIT system can be improved by choosing the correct drive pattern, which is a strategy that selects the electrode pairs on which current injection and voltage readings are performed. In addition, some studies [16,17] have analyzed the amount of information that these current and measurement patterns can convey.

Therefore, even if the general topic of discussing which drive pattern is the optimal one to choose in EIT applications has been studied in the past, none have examined the relationship between the response of an EIT system and the contact position of the input stimuli. For this reason, in [18], we have preliminarily investigated how the boundary voltage data in an EIT sensor were affected by the target position using an 8-electrodes prototype system.

In this paper, we present our work on optimizing the selection of drive pattern based on the input stimulus position. Indeed, we aim to further improve EIT sensor systems by introducing a more complete analysis on their performances. We begin by presenting our 16-electrode EIT system that allows us to study the characteristics of three drive patterns. We then introduce three performance parameters that can be used to quantitatively evaluate the reconstructed images, in terms of target size detection and position accuracy. These performance parameters are independent of data collection rate, current amplitude, frequency, drive pattern strategy, and number of contact positions. Thus, they can be used to specify the performance of any EIT-based sensor. We conduct our study in the case of single and multiple input contact positions using both simulated and experimental EIT voltage data.

The novelty of this work consists of demonstrating that target size detection and position accuracy in EIT-tactile sensors can be improved by switching between different drive patterns strategies based on the contact position of the target.

This approach could potentially result in a first step to surmount the major drawbacks of these sensors.

In fact, our results show that each drive pattern responds differently for different potions of the input target, influencing the overall performance of the EIT-sensor.

The remainder of this paper is organized as follows: The EIT mathematical formulation and our application on EIT-based pressure sensors is discussed in Section 2. In Section 3, we begin by presenting the performance parameters for evaluating EIT-based sensors, and we then describe the drive patterns studied in this paper. The model of the sensor is shown along with simulation studies and the experimental setup. In Section 4, we show and discuss the experimental results and the different images corresponding to pressure inputs on the stretchable sensor. Finally, Section 5 concludes the paper.

## 2. Electrical Impedance Tomography

### 2.1. EIT Mathematical Formulation

In conventional X-ray or computed tomography procedures, a high-frequency signal is applied to the body under examination. In contrast to these methods, EIT can be seen as a low frequency electrical imaging technique, where the induced magnetic field can be neglected, as discussed in [12]. For this reason, EIT is used to infer the internal changes in conductivity of the body that are affecting the surface potential measurements. In order to do so, electrodes are located around the periphery of the electrically conductive body and current is applied between two of them. Reconstruction techniques are then used to show the conductivity changes inside the body.

EIT is structured in two sub-categories: in the forward model, an initial conductivity of the body is assumed and then the voltage data at the electrodes are calculated by solving the Laplacian elliptic partial differential equation. For a given body Ω with conductivity σ, the scalar potential is ϕ and the electric field E=−∇ϕ. We describe the steady-state absence of interior current sources as:

(1) ∇·σ∇ϕ=0.

The analysis [12] is completed with the boundary equations at the electrodes; it is commonplace to use the complete electrode model:

$$\sigma \nabla \phi \cdot n=\frac{1}{z_{l}}\left(V_{l}-\phi \right),$$

∫σ∇ϕ·n∂Ω=0,

$$\sum_{l=1}^{L}I_{l}=0,$$

$$\sum_{l=1}^{L}V_{l}=0,$$

where $V_{l}$ and $I_{l}$ are, respectively, the potential and the current at the *l*’th electrode, $z_{l}$ is the contact impedance between the *l*’th electrode and the domain, and n is the unit normal to the periphery of the body. These differential equations are implemented by computing a Finite Element (FE) model of the system.

The second sub-category of the EIT problems is the inverse approach. Here, the unknown conductivity distribution is reconstructed by using the measured voltage data. Mathematically, this is done by minimizing the sum of squared residuals:

(2) $$\begin{array}{c} min(||V_{m}-F\left(\sigma \right)|{|}^{2}), \end{array}$$

where $V_{m}$ is the data set containing the potentials measured at the electrodes. F(σ) is the conductivity distribution to be recovered through the forward operator F(.) that maps this conductivity to the potentials at the boundary. The next step is the linearization of F(σ) around the assumed initial estimation of the conductivity $\sigma_{0}$:

$$\;V_{m}-F\left(\sigma_{0}\right)-\frac{\partial F(\sigma_{0})}{\partial \sigma }\left(\sigma -\sigma_{0}\right)=0,$$

δV≈Jδσ,

(3) $$\begin{array}{c} \delta \sigma =J^{-1}\delta V. \end{array}$$

$V_{m}-F\left(\sigma_{0}\right)=\delta V$ represents the variation in the measured potentials when a conductivity change has taken place and *J* is the Jacobian matrix. *J* is calculated using a discretization of the internal conductivity. In this work, we determined *J* by adopting the calculations shown in [19], assuming a small conductivity perturbation into the FE mesh, and then solving the forward problem to the boundary changes in potential δV. There are several alternative approaches for the calculation of *J*. The reader is referred to [12,20] for exhaustive reviews of them. Many entries in the Jacobian matrix have values close to zero, which creates high sensitivity to small changes in δV; this ill-conditioned problem is solved through regularization.

### 2.2. Regularization

Tikhonov regularization is commonly used in EIT applications. Regularization means finding a trade-off between the “exact” but unstable solution based on the measured data, and a more stable “approximate” solution controlled by an imposed prior. Here, the prior information is the assumption of smoothness of the spatial distribution of δσ. As a consequence, the spatial resolution decreases, as well as and the ability to reconstruct sharp changes in the conductivity. The formal solution through Tikhonov regularization is:

(4) $$\begin{array}{c} \delta \sigma ={\left(J^{T}J+\alpha^{2}R^{T}R\right)}^{-1}\left(J^{T}\delta V\right),\; \end{array}$$

where α is a scalar hyperparameter that controls the amount of regularization and *R* is a regularization matrix that controls the “smoothness” of the solution. Different algorithms can be used for the choice of α and *R*. In this work, we have employed NOSER, an acronym for Newton’s One-Step Error Reconstructor prior [21]. This algorithm is based on the methods of least squares, and the regularization matrix is scaled by the sensitivity *s* of each element:

(5) $$\begin{array}{c} R^{T}R=diag{\left[J^{T}J\right]}^{s}, \end{array}$$

where s∈[0,1].

### 2.3. Image Reconstructions

**Figure 1:**
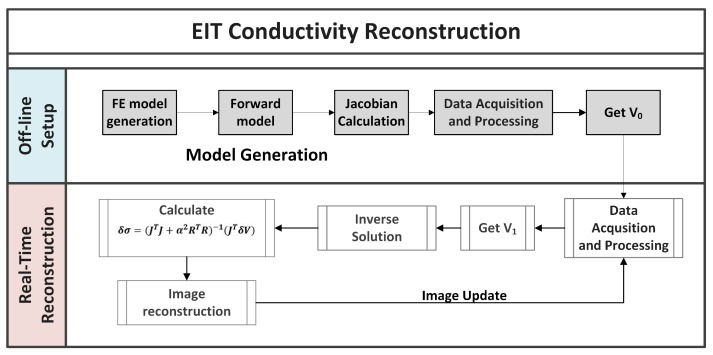
Flow chart of Electrical Impedance Tomography (EIT) image reconstruction. The grey shaded boxes show the calculations done in the offline setup of the system. Then, the second set of potentials $V^{1}$ is updated online and an image showing the conductivity changes inside the sensor is reconstructed.

As shown in Figure 1, all the parameters for inverse solutions are computed offline. The image of the pressure contacts over the sensor is then reconstructed by comparing two voltage data sets: $V^{0}=({V^{0}}_{1},\ldots ,{V^{0}}_{k})$ indicates the background set of voltages used as a reference and $V^{1}=({V^{1}}_{1},\ldots ,{V^{1}}_{k})$ the boundary voltages measured after a conductivity change takes place due to a pressure input over the sensor. *k* is the number of measurements. $V^{0}$ is obtained only once in the offline system setup and then the second set of potentials $V^{1}$ is updated in real-time. EIT systems usually achieve a temporal frequency of around 40 Hz [22] or more [23].

In our case, the image reconstruction was carried out using an open source MATLAB toolkit (Electrical Impedance and Diffuse Optical tomography Reconstruction Software (EIDORS) software algorithm [24]).

## 3. Methods

### 3.1. Performance Parameters

Here, we propose a methodology for EIT image and data quality analysis. We present a set of evaluation parameters, taking inspiration from the works of [13,25], and apply them in the case of an EIT-based sensor. These parameters will be used to define the performance of the sensor and compare the results when three drive patterns are used for different target locations. The performance parameters are independent of data collection rate, current amplitude, frequency, drive pattern strategy, and number of contact positions. As a result, our proposed approach can be used to specify the performance of any EIT-based sensor.

The first two parameters are used to study data accuracy of the boundary voltage. Then, we present the image quality parameters that want to characterize the exactness of the reconstructed images, detectability and distinguishability of the targets.

#### 3.1.1. Voltage Data Parameters

In EIT systems, Signal-to-Noise Ratio (SNR) is used to judge the quality of the signal and precision of measurements as seen in [26,27]. It is a ratio between the desired signal and the level of the unwanted background noise. The formula is:

(6) $$\begin{array}{c} \mathrm{SNR}=-20\log_{10}\frac{\left|E[V_{i}]\right|}{\sqrt{Var(V_{i})}}, \end{array}$$

where $E[V_{i}]$ is the mean of multiple measurements on each channel and $Var(V_{i})$ the variance between these measurements.

The most well-known approach to optimizing current injections is based on the distinguishability criterion, as shown by Isaacson [28]. Here, we consider that it would be ideal to maximize the norm of the difference between the electrode potentials corresponding to an unknown and known reference conductivity distribution. Therefore, the Boundary Voltage Changes (BVC) parameter is found as:

(7) $$\begin{array}{c} \mathrm{BVC}=\left|\right|V^{1}-V^{0}\left|\right|. \end{array}$$

#### 3.1.2. Image Parameters

After the image $\left(\hat{x_{O}}\right)$ of the conductivity changes is reconstructed, it needs to be post-processed. However, the image contains artifacts due to noise, electrodes movement and the EIT inverse problem. To minimize these effects, we work on the image pixel values ${\left[\hat{x_{O}}\right]}_{i}$ and select the area (Region Of Interest, ROI) in which the maximum amount of conductivity change has taken place. The processed image ($\hat{x_{P}}$) is found as follows:

(8) $$\begin{array}{c} {\hat{\left[x_{P}\right]}}_{i}=\left\{\begin{array}{cc} {\left[\hat{x_{O}}\right]}_{i}, & \mathrm{if}{\left[\hat{x_{O}}\right]}_{i}\geq f\cdot max\left(\hat{x_{O}}\right),\\ 0, & \mathrm{otherwise}, \end{array}\right. \end{array}$$

where ${\hat{\left[x_{P}\right]}}_{i}$ are the pixel values of ($\hat{x_{P}}$), and *f* is the threshold for the selection. The ROI is the region of ($\hat{x_{O}}$), where the pixels of ($\hat{x_{P}}$) are non zero.

The performance parameters for assessing the quality of the reconstructed image are presented here and will be used in the case of single and simultaneous contact positions.

**Figure 2:**
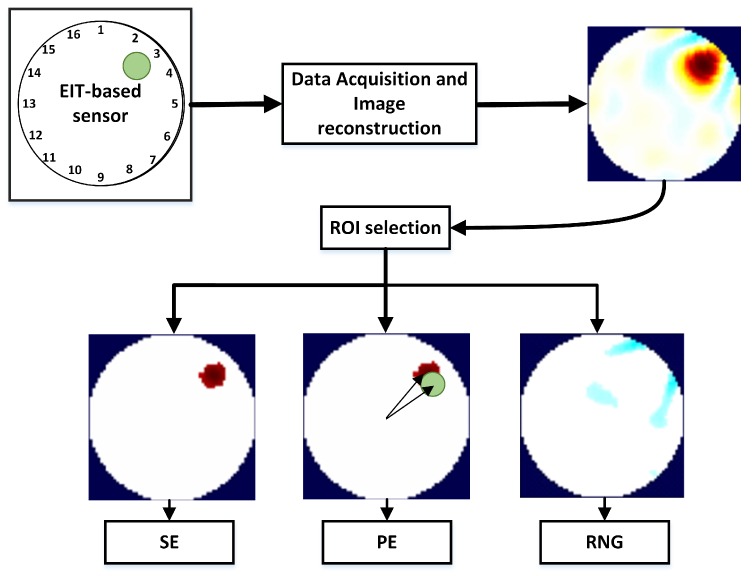
Performance parameters for the reconstructed image. On the top left, the EIT-based sensor, and in green the target placed over it. After the voltage data is acquired, the image of the conductivity change is reconstructed (top right, $\left(\hat{x_{O}}\right)$). Then, the Region Of Interest (ROI) is selected and the parameters of Size Error (SE), Position Error (PE) and Ringing (RNG) are calculated from the post-processed image $\left(\hat{x_{P}}\right)$.

Size Error (SE) measures the difference between the Detected (DSO) and the real (SO) Size of the Object compared to the Area of the entire Conductive Medium ($A_{CM}$):

(9) $$\begin{array}{c} \mathrm{SE}=\left|\frac{\mathrm{DSO}-\mathrm{SO}}{A_{CM}}\right|, \end{array}$$

where the DSO is obtained by calculating the number of pixels in the ROI (Figure 2).

Position Error (PE) (Figure 2) shows the mismatch between the detected position of the object and the real one. It is found as:

(10) $$\begin{array}{c} \mathrm{PE}=\left|r_{r}-r_{d}\right|, \end{array}$$

where $r_{r}$ and $r_{d}$ indicate the real and estimated positions of the object, respectively. Since $r_{r}$ and $r_{d}$ are found by extracting the centroid of the pixels contained in the ROI, the PE parameter does not depend significantly on the choice of the threshold factor *f*.

Figure 2 shows an arc of light blue ringing around the ROI. Ringing (RNG) measures whether reconstructed images show bands or “ghosts” of the opposite sign surrounding the main reconstructed target area. Ringing is typical of linear filters, caused by the overshoot-undershoot behavior of the system. The conductivity change creates an overshoot in its values. Then, the response bounces back below the steady-state level, causing the first ring, and then oscillates back and forth above and below the steady-state level. RNG is calculated by measuring the area of opposite sign $A_{INV}$ surrounding the reconstructed target:

(11) $$\begin{array}{c} \mathrm{RNG}=\frac{A_{INV}}{A_{CM}-\mathrm{DSO}}. \end{array}$$

Best values of RNG are low and uniform because ringing artifacts might interfere with the detection of contact points.

### 3.2. Drive Patterns

As already mentioned, a drive pattern is a strategy that selects the electrodes pairs on which current injection and voltage reading are performed. For each drive pattern, two electrodes are chosen for the current injection while the other two are used for voltage measurement; this process is systematically repeated until every electrode pair has served for the current injection. The electrodes carrying current are generally not used for voltage measurement in order to reduce the effects of noise from the electrode contact impedance. In addition, due to the reciprocity principle [29], not all the voltage measurements are independent between each other, reducing the total number of information available for the image reconstruction.

**Figure 3:**
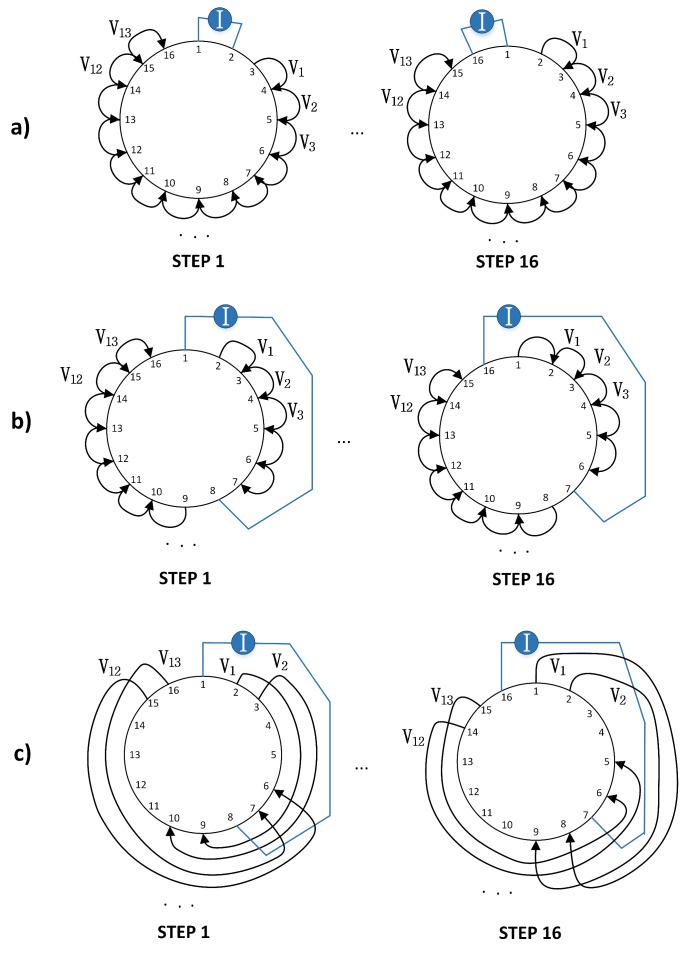
From top to bottom, the first and last of sixteen steps for the (**a**) Adjacent (AD); (**b**) Pseudo-Polar (PP) and (**c**) PP-PP drive patterns for a 16-electrode system. For each injection step, current is applied between a pair of electrodes and the resulting voltage is measured between the remaining pairs. The current excitation and voltage measurement is then rotated until the last step.

For this study, we have selected three drive patterns: 

Adjacent drive pattern (AD): in the AD in Figure 3a, the driving current is injected between adjacent electrodes and the potentials are read on the remaining electrodes pairs. The AD pattern is the one that has been used mostly in EIT applications.

Pseudo-Polar (PP) drive pattern: the PP drive pattern (in Figure 3b) uses almost the opposite drive electrodes for current injection. The voltage readings are then measured through neighbouring electrodes.

PP-PP drive pattern: In this study, we are presenting the PP-PP drive pattern in Figure 3c. This drive pattern presents drive and reading electrodes with the same distribution of electrodes as when performing PP current injection. The capability of exciting and reading ample sections of the material could be useful in the case of distributed pressure points. To the best of our knowledge, none have ever presented and studied in the EIT literature the behaviour of this drive pattern. 

Our choice for the above drive patterns was mainly based on the higher number of independent measurements compared to the others found in the literature. In fact, these drive patterns have a total number of independent measurements equal to: k=L(L−3)/2, where *L* is the number of electrodes. For our 16-electrode sensor system, k=104. A detailed description of the number of measurements in EIT can be found in [30].

Other types of drive patterns mainly used in EIT systems include trigonometric [31] and polar (opposite) [32] patterns. In polar patterns, the electrodes injecting current are 180° apart. This approach increases the current density inside the material, but also reduces the number of independent measurements and therefore the amount of information available for the conductivity reconstruction. Trigonometric patterns, on the other hand, show less tolerance to errors. This behavior was studied in Kolehmainen et al. [33]. In trigonometric patterns in fact, voltages are measured on the current-carrying electrodes, which creates a high sensitivity to contact impedance mismatches. For the above reasons, we have limited the drive patterns studied in this work to the AD, PP and PP-PP patterns.

### 3.3. Sensor Model

Following the works of [34,35], we have modelled our conductive fabric as an electric circuit network with a certain number of length-related and contact resistors. When pressure is applied over the fabric material, the gap between the conductive yarns decreases (i.e., the contact area between the knitting courses increases) so there is a reduction in the overall resistance in that area. As a consequence, the current density in the area of the input pressure will increase.

Following this model, the AD pattern is expected to produce an improved image reconstruction when a pressure input is applied close to the electrodes, as the current is mainly flowing in that area.

In the PP pattern, the current density is higher across the centre of the conductive medium. Therefore, this drive pattern will show a better reconstruction when the pressure input is more central.

Lastly, in the PP-PP pattern, the current distribution is the same as the PP pattern, but the portion of the material being read is higher. This drive pattern is then expected to result in an improved performance when the pressure inputs are more distributed, as in the case of multiple contact locations.

### 3.4. Experiments

**Figure 4:**
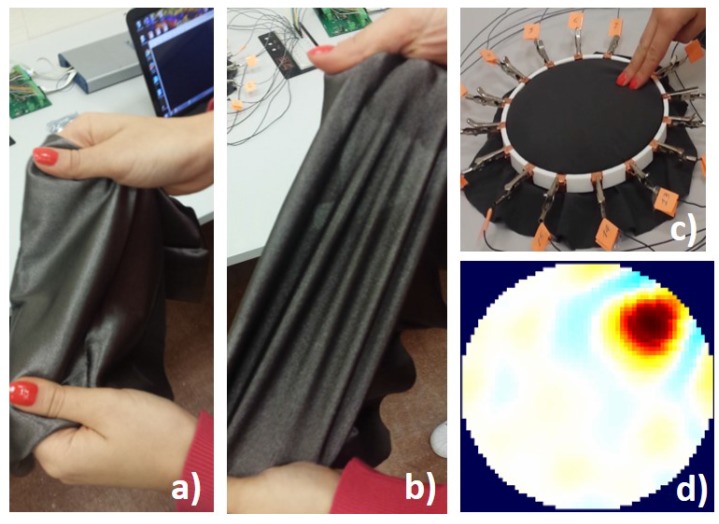
Our EIT-based stretchable sensor. In (**a**), the conductive fabric material in shown; and, in (**b**), the material when stretched; in (**c**), a touch pressure is applied over the sensor and, in (**d**), an image showing the conductivity change is reconstructed from the boundary voltage data. Red colour indicates a positive changes in the conductivity.

In our work, we have used a piezoresistive, nylon stretchable fabric from Eeonyx Corporation (www.eeonyx.com) made of conductive doped polypyrrole that responds to pressure with local changes in its conductivity. The material is low-cost, light weight and can be stretched several times its original length without compromising its conductivity (Figure 4a,b).

Our experimental set-up consists of a 3D-printed circular frame made of two disc layers used to house the conductive sheet. The frame presents 16 extrusions where conductive copper stripes are fixed to create the electrodes. The conductive fabric is placed between the two discs, firmly in contact with the surrounding electrodes, as shown in Figure 4c.

**Figure 5:**
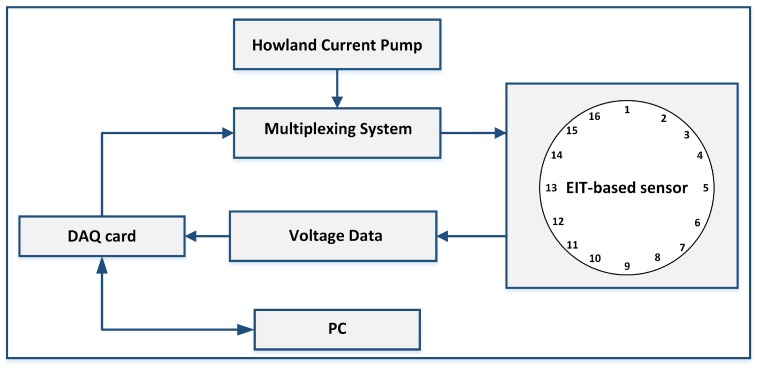
Block diagram of EIT hardware system for current injection and voltages measurement.

A block diagram of our hardware system for the current injection and voltages measurement is shown in Figure 5. It consists of our customized Printed Circuit Board (PCB) presenting a Howland Current Pump constant current generator and two multiplexers for the rotation of the current supply between the electrodes at each time step. The multiplexers are digitally-controlled by a National Instruments Data Acquisition (NI DAQ) USB card. The Howland Current Pump provides a constant DC current supply, independent from the connected load resistivity. Using a DC current for EIT-based sensors applications is advisable [5]. In fact, the use of a DC current excitation is advantageous because of possible future implementation of EIT-sensors in battery-powered wearable applications.

This hardware design guarantees a voltage data set collection rate higher than 70 Hz.

**Figure 6:**
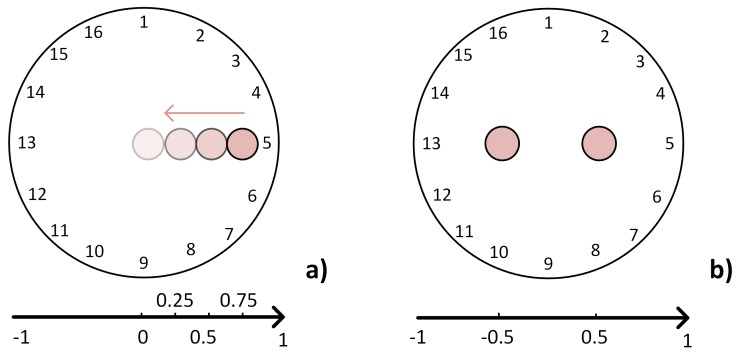
In (**a**), the experiments are conducted with the load applied each time step at x=0.75, x=0.5, x=0.25 and finally at x=0. The last experiment in (**b**) is the simultaneous two-point pressure at locations x1=−0.5 and x2=0.5.

For our analysis, five different scenarios have been considered, where each scenario has been tested with the three drive patterns. Figure 6 shows the four targets in the case of one-point input location and the scenario with two-point inputs. A non-conductive paper grid is placed over the sensor to perform exact target placements. A load of 1.46% the size of the conductive medium is applied during each test in different locations along the *x*-axis from left to right until the centre of the conductive medium. In this study, experiments with different loads are not reported. In fact, we have found that the load itself does not change the performance parameters for each drive pattern as significantly as the location of the input target.

Various studies have been conducted on finding the correct choice of the threshold factor [5,36], but heuristic selection is still very common. Nevertheless, to post-process the image and choose a threshold factor is a necessary step to detect size and position of the object in contact with the sensor. In our case, we have chosen the threshold factors as *f*= 0.10, 0.08 and 0.05 for drive patterns AD, PP and PP-P, respectively. The above values are the ones that were on average performing the best in terms of size detection. In fact, as explained in Section 3.1.2, the Position Error parameter does not depend on the choice of *f*. Different drive patterns behave differently and using a post-processing techniques is needed to fairly compare the performance of one drive pattern over another when studying their dependency on the input target position, as in the case of this study.

### 3.5. Simulation Studies

For carrying out simulations, we have considered a 16-electrode circular shaped phantom that models our sensor system. An FE mesh structure with 5184 elements has been used to solve the forward and inverse problems. The targets have been generated by simulating a pressure input for each experiment, as described in Section 3.4. The simulated boundary voltages consist of V0 and V1, before and after a conductivity change has taken place. These data sets are then used to reconstruct the image of the conductivity change due to the simulated pressure over the sensor. The performance parameters are then calculated from each reconstructed images.

**Figure 7:**
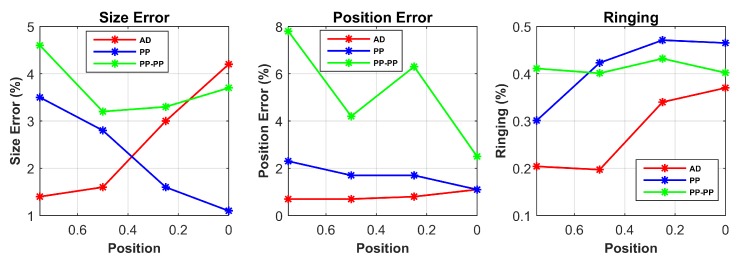
Performance parameters from simulated data are shown in the case of single point input positions.

Table 1 summarizes the response of SE, PE and RNG for different target locations for each drive pattern from the simulated data. The SE and PE parameters are shown in % relatively to the size of the sensor. For better visualization, the results in the case of single point contact location are additionally shown in Figure 7.

## 4. Experimental Results and Discussion

### 4.1. Voltage Data Parameters

Table 2 shows the SNR for the three drive patterns. As expected, the PP-PP drive pattern produces the highest signal compared to noise since the current flowing crosswise the sensor is increased and the readings are taken over ample sections of the material.

The BVCs for the three drive patterns are shown in Table 3. A drive pattern with small BVC is more likely to be negatively influenced by the presence of noise. From the results, we can see that the BVCs for the AD pattern present small values, confirming the work of [32]. The PP and PP-PP patterns present higher BVCs, thus will be privileged in the case of noisy systems. These results follow our sensor model and show that all the patterns have higher BVCs in the case of two-point contact locations, as a result of the increased conductivity change inside the medium.

### 4.2. Image Parameters

**Figure 8:**
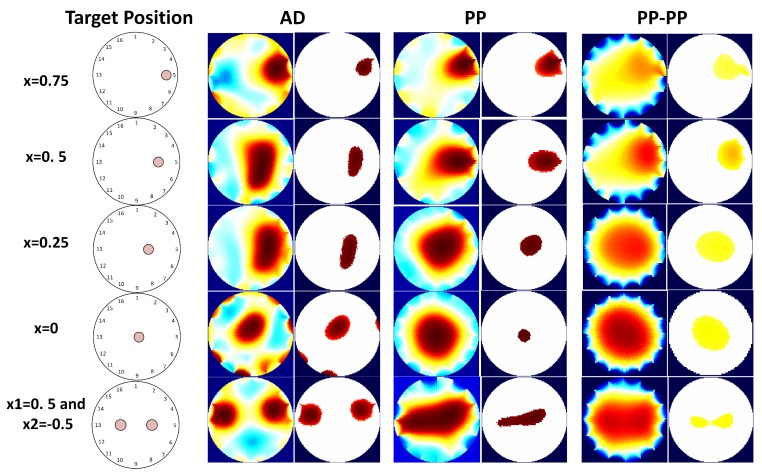
On the left, the real contact locations are shown, and for each one the reconstructed and processed images in the case of AD, PP and PP-PP pattern are presented.

**Figure 9:**
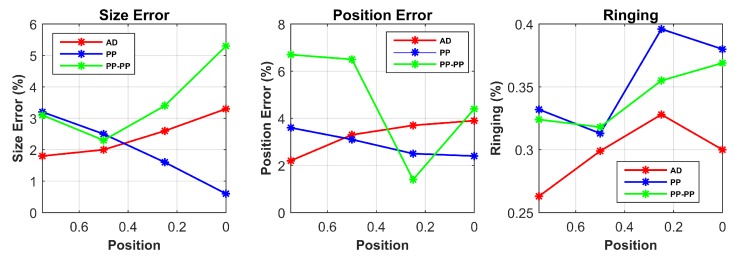
Performance parameters from experimental data are shown in the case of single point input positions.

Figure 8 shows the reconstructed images from the experimental data. These images are used to calculate the performance metrics in Table 4. The experimental results are in line with the simulations and follow our predictions from the sensor model. For better visualization, the results for single point contact location are also plotted in Figure 9.

The first result to consider is that the target position does not influence the RNG parameter. Since ringing effect is due to the linear filter behaviour of the system, it is mainly affected by the drive pattern choice. However, it is worth noticing that RNG is always reasonably uniform and low for the AD pattern in all scenarios, while its higher values for both PP and PP-PP patterns might result in an incorrect interpretation of the reconstructed image.

In both simulation and experimental tests, the AD pattern performs better for SE and PE when the contact position is close to the electrodes; in addition, compared to the other patterns, the PE is less variable at different positions, making the interpretation of the results more reliable.

The PP pattern presents a good response for SE and PE when the target is placed in the central areas of the conductive medium (x=0.25 and x=0). This demonstrates its improved performances when compared to the other patterns in the same scenarios, thus its good sensitivity in the centre of the domain.

Lastly, when compared to the other drive patterns, PP-PP performs better in the presence of two simultaneous contact positions. In the other cases, the response of this drive pattern is quite poor. In addition, this pattern does not show any dependency between the position of the target and its performance. This behaviour makes the PP-PP pattern unfeasible and unstable for all scenarios but simultaneous contact locations.

### 4.3. Discussion

To sum up, both simulation and experimental results show that the AD pattern would be preferable in the case of contact positions close to the electrodes. The reason is that this strategy gives the highest current density in those areas, so the conductivity changes will be more easily detected. In addition, its response is more reliable in terms of PE, although the BVCs show that this pattern would not perform well in the case of noisy systems.

In the PP pattern, by making the current injection from electrodes that are almost 180° apart, the current density into the sensor is increased. As a result, this pattern performs better in the case of contact location in the centre of the sensor.

Finally, the capability of reading ample sections of the material makes the PP-PP pattern strategy useful in the case of multiple pressure points, as the conductivity change due to pressure is more distributed.

These results are also confirmed by Figure 8, which shows that the three drive patterns have different responses for the same target position.

As a consequence of these results, we believe that, in order to improve target size detection and position accuracy of an EIT sensor, it would be desirable to switch in real time between the AD, PP or PP-PP patterns depending on the position of the target input over the sensor. This should be done immediately upon the first detection of the target.

The feasibility of this approach is reinforced by the high temporal resolution of EIT systems, which has been discussed in Section 2.3. In addition, in Section 3.4, we have shown our experimental setup presenting a data set collection rate higher than 70 Hz. This guarantees a switching time between drive patterns in less than 14 ms, which will only slightly affect the real-time reconstruction speed of the sensor and will improve its target size detection and position accuracy.

## 5. Conclusions

The contribution of this study was to propose a method that aims to improve the performance of EIT-based sensors. This can be performed by selecting the correct drive pattern based on the input stimulus position.

We have conducted a comparative analysis on the performance of three drive patterns in the case of different target locations over the EIT-sensor. The response of the drive patterns was evaluated using performance parameters on simulation and experimental data from our 16-electrode EIT-based pressure sensor.

Based upon the results, both simulations and experiments confirmed our hypothesis of a certain amount of dependency between the position of the input pressure over the EIT-based sensor and the performance of the selected drive pattern. The results show that: (a) although the AD pattern is less tolerant to noise and has a lower dynamic range, it performs better when the target is placed close to the electrodes; (b) the PP pattern is preferable when the target is close to the central area of the sensor; and (c) the PP-PP pattern is suitable for the detection of simultaneous contact pressures only.

In fact, the target size detection and position accuracy of the sensor may improve up to 4.7% and 18% when choosing the correct drive pattern over another based on the target location. This improvement can be also visualized in the reconstructed images of the pressure inputs and confirms our hypothesis derived from the model of the sensor. Such improvements may help to surmount the major limitations of EIT-based sensors and enable their potential applications, considering the promising advantages of using such sensors.

The above results were obtained by using a different threshold factor for each drive pattern. The choice of *f* was based on using the one that was best performing in terms of Size Detection, so that the drive patterns could be compared when performing at their best. The choice of *f* was therefore a necessary step to compare the performance of the drive patterns. Future research needs to investigate a method to automatically select *f* in order to make EIT studies more objective and rigorous.

Furthermore, we are currently working on evaluating the amplitude response in relation to the applied pressure, as EIT-based sensors show poor ability to discriminate between pressure intensities. Experiments need to be conducted with simultaneous different loads and target dimensions.

## Figures and Tables

**Table 1:** Performance parameters from simulated data.

Position	AD			PP			PP-PP		
	SE	PE	RNG	SE	PE	RNG	SE	PE	RNG
x=0.75	1.4%	0.7%	0.204	3.5%	2.3%	0.301	4.6%	7.8%	0.411
x=0.5	1.6%	0.7%	0.197	2.8%	1.7%	0.423	3.2%	4.2%	0.401
x=0.25	3.0%	0.8%	0.340	1.6%	1.7%	0.471	3.3%	6.3%	0.432
x=0	4.2%	1.1%	0.370	1.1%	1.1%	0.465	3.7%	2.5%	0.402
x1=0.5 and x2=0.5	4.4%	23.4%	0.451	2.5%	22.6%	0.472	1.5%	14.4%	0.428

**Table 2:** Signal-to-Noise Ratio (dB) for the three different drive patterns.

AD	PP	PP-PP
54.98	58.93	76.06

**Table 3:** Boundary voltage changes (mV) for the three different drive patterns at different target locations.

Position	AD	PP	PP-PP
x=0.75	55	182	587
x=0.5	52	156	900
x=0.25	49	273	547
x=0	16	368	697
x1=0.5 and x2=0.5	129	1324	1487

**Table 4:** Performance parameters from experimental data.

Position	AD			PP			PP-PP		
	SE	PE	RNG	SE	PE	RNG	SE	PE	RNG
x=0.75	1.8%	2.2%	0.263	3.2%	3.6%	0.332	3.1%	6.7%	0.324
x=5	2.0%	3.3%	0.299	2.5%	3.1%	0.313	2.3%	6.5%	0.318
x=0.25	2.6%	3.7%	0.328	1.6%	2.5%	0.396	3.4%	1.4%	0.355
x=0	3.3%	3.9%	0.300	0.6%	2.4%	0.380	5.3%	4.4%	0.369
x1=0.5 and x2=0.5	1.7%	38.0%	0.353	1.3%	22.5%	0.372	1.0%	20.0%	0.361

## References

[1] Smith R.W., Freeston I.L., Brown B.H. (1995). A real-time electrical impedance tomography system for clinical use-design and preliminary results. IEEE Trans. Biomed. Eng..

[2] Bodenstein M., David M., Markstaller K. (2009). Principles of electrical impedance tomography and its clinical application. Crit. Care Med..

[3] Tallman T., Gungor S., Wang K., Bakis C. (2014). Damage detection and conductivity evolution in carbon nanofiber epoxy via electrical impedance tomography. Smart Mater. Struct..

[4] Knight R., Lipczynski R. The use of EIT techniques to measure interface pressure. Proceedings of the Twelfth Annual International Conference of the IEEE Engineering in Medicine and Biology Society.

[5] Silvera-Tawil D., Rye D., Soleimani M., Velonaki M. (2015). Electrical impedance tomography for artificial sensitive robotic skin: A review. IEEE Sens. J..

[6] Wang H., de Boer G., Kow J., Alazmani A., Ghajari M., Hewson R., Culmer P. (2016). Design Methodology for Magnetic Field-Based Soft Tri-Axis Tactile Sensors. Sensors.

[7] Tomo T.P., Somlor S., Schmitz A., Jamone L., Huang W., Kristanto H., Sugano S. (2016). Design and characterization of a three-axis hall effect-based soft skin sensor. Sensors.

[8] Cirillo A., Ficuciello F., Natale C., Pirozzi S., Villani L. (2016). A Conformable Force/Tactile Skin for Physical Human–Robot Interaction. IEEE Robot. Autom. Lett..

[9] Silvera Tawil D., Rye D., Velonaki M. (2012). Interpretation of the modality of touch on an artificial arm covered with an EIT-based sensitive skin. Int. J. Robot. Res..

[10] Nagakubo A., Alirezaei H., Kuniyoshi Y. A deformable and deformation sensitive tactile distribution sensor. Proceedings of the IEEE International Conference on Robotics and Biomimetics, 2007 (ROBIO 2007).

[11] Tallman T., Gungor S., Wang K., Bakis C. (2015). Tactile imaging and distributed strain sensing in highly flexible carbon nanofiber/polyurethane nanocomposites. Carbon.

[12] Holder D.S. (2004). Electrical Impedance Tomography: Methods, History and Applications.

[13] Xu C., Dong X., Shi X., Fu F., Shuai W., Liu R., You F. Comparison of drive patterns for single current source EIT in computational phantom. Proceedings of the 2nd International Conference on Bioinformatics and Biomedical Engineering, 2008, (ICBBE 2008).

[14] Gallo G.J., Thostenson E.T. (2016). Spatial damage detection in electrically anisotropic fiber-reinforced composites using carbon nanotube networks. Compos. Struct..

[15] Demidenko E., Hartov A., Soni N., Paulsen K.D. (2005). On optimal current patterns for electrical impedance tomography. IEEE Trans. Biomed. Eng..

[16] Kaipio J.P., Seppänen A., Voutilainen A., Haario H. (2007). Optimal current patterns in dynamical electrical impedance tomography imaging. Inverse Probl..

[17] Silva O.L., Lima R.G., Martins T.C., de Moura F.S., Tavares R.S., Tsuzuki M.S.G. (2017). Influence of current injection pattern and electric potential measurement strategies in electrical impedance tomography. Control Eng. Pract..

[18] Russo S., Carbonaro N., Tognetti A., Nefti-Meziani S. (2017). A Quantitative Evaluation of Drive Patterns in Electrical Impedance Tomography. Lecture Notes of the Institute for Computer Sciences, Social Informatics and Telecommunications Engineering, Proceedings of the 6th EAI International Conference on Wireless Mobile Communication and Healthcare, Milan, Italy, 14–16 November 2016.

[19] Polydorides N., Lionheart W.R. (2002). A Matlab toolkit for three-dimensional electrical impedance tomography: A contribution to the Electrical Impedance and Diffuse Optical Reconstruction Software project. Meas. Sci. Technol..

[20] Brandstatter B. (2003). Jacobian calculation for electrical impedance tomography based on the reciprocity principle. IEEE Trans. Magn..

[21] Lionheart W.R. (2004). EIT reconstruction algorithms: Pitfalls, challenges and recent developments. Physiol. Meas..

[22] Alirezaei H., Nagakubo A., Kuniyoshi Y. A highly stretchable tactile distribution sensor for smooth surfaced humanoids. Proceedings of the 7th IEEE-RAS International Conference on Humanoid Robots, 2007.

[23] Wilkinson A.J., Randall E., Cilliers J., Durrett D., Naidoo T., Long T. (2005). A 1000-measurement frames/second ERT data capture system with real-time visualization. IEEE Sens. J..

[24] Adler A., Lionheart W.R. (2006). Uses and abuses of EIDORS: An extensible software base for EIT. Physiol. Meas..

[25] Yasin M., Böhm S., Gaggero P.O., Adler A. (2011). Evaluation of EIT system performance. Physiol. Meas..

[26] Gagnon H., Cousineau M., Adler A., Hartinger A.E. (2010). A resistive mesh phantom for assessing the performance of EIT systems. IEEE Trans. Biomed. Eng..

[27] Bera T.K., Nagaraju J. (2012). Studying the resistivity imaging of chicken tissue phantoms with different current patterns in Electrical Impedance Tomography (EIT). Measurement.

[28] Isaacson D. (1986). Distinguishability of conductivities by electric current computed tomography. IEEE Trans. Med. Imaging.

[29] Geselowitz D.B. (1971). An application of electrocardiographic lead theory to impedance plethysmography. IEEE Trans. Biomed. Eng..

[30] Brown B., Seagar A. (1987). The Sheffield data collection system. Clin. Phys. Physiol. Meas..

[31] Cheney M., Isaacson D., Newell J.C., Simske S., Goble J. (1990). NOSER: An algorithm for solving the inverse conductivity problem. Int. J. Imaging Syst. Technol..

[32] Shi X., Dong X., Shuai W., You F., Fu F., Liu R. (2006). Pseudo-polar drive patterns for brain electrical impedance tomography. Physiol. Meas..

[33] Kolehmainen V., Vauhkonen M., Karjalainen P., Kaipio J. (1997). Assessment of errors in static electrical impedance tomography with adjacent and trigonometric current patterns. Physiol. Meas..

[34] Zhang H., Tao X., Yu T., Wang S. (2006). Conductive knitted fabric as large-strain gauge under high temperature. Sens. Actuators A Phys..

[35] Li L., Au W.M., Wan K.M., Wan S.H., Chung W.Y., Wong K.S. (2010). A resistive network model for conductive knitting stitches. Text. Res. J..

[36] Naushad A., Rashid A., Mazhar S. Analysing the performance of EIT images using the point spread function. Proceedings of the 2014 International Conference on Emerging Technologies (ICET).

